# Expression and Functional Relevance of Death-Associated Protein Kinase in Human Drug-Resistant Epileptic Brain: Focusing on the Neurovascular Interface

**DOI:** 10.1007/s12035-018-1415-z

**Published:** 2018-11-09

**Authors:** Sherice Williams, Mohammed Hossain, Saurabh Mishra, Jorge Gonzalez-Martinez, Imad Najm, Chaitali Ghosh

**Affiliations:** 1 Cerebrovascular Research, Department of Biomedical Engineering (ND 20), Lerner Research Institute, Cleveland Clinic, 9500 Euclid Avenue, Cleveland, OH 44195 USA; 2 Epilepsy Center, Neurological Institute, Cleveland Clinic, Cleveland, OH USA; 3 Department of Molecular Medicine, Cleveland Clinic Lerner College of Medicine of Case Western Reserve University, Cleveland Clinic, Cleveland, OH USA

**Keywords:** Biomarker, Drug resistance, Death receptor, Blood-brain barrier, Angiogenesis, Hypoxia

## Abstract

Death-associated protein kinase (DAPK) is a key player in various cell death signaling pathways. Prolonged seizures induce neuronal stress; thus, we studied DAPK expression in resected brain tissues from patients with refractory epilepsy and the pathophysiological relevance of neurovascular DAPK. We used brain resections from temporal lobe epilepsy (TLE), tumor (BT), arteriovenous malformation (AVM), and autopsy, and isolated human endothelial cells (EPI-ECs) and glial cells (EPI-Astro) from epileptic brains compared to control brain endothelial cells (HBMECs) and astrocytes. DAPK and phosphorylated DAPK (p-DAPK) expression was evaluated by immunohistochemistry and western blot. Subcellular localization of DAPK in epileptic brain was explored; DAPK mRNA/protein levels in EPI-ECs/EPI-Astro were evaluated. We assessed DAPK localization with hypoxic inducible factor (HIF-1α) and vascular endothelial growth factor (VEGF) in epilepsy, BT, and AVM. We found DAPK overexpression across neurons, microcapillaries, and astrocytes in TLE vs controls; DAPK and p-DAPK levels significantly increased only in microsomal fractions of epileptic brain. DAPK mRNA remained unchanged, although increased DAPK and p-DAPK protein expression was observed in EPI-ECs. DAPK inhibition reduced p-DAPK, HIF-1α, and VEGF expression, but increased cytotoxicity and decreased cell viability in EPI-ECs and EPI-astro vs. controls. DAPK staining in TLE resembled BT and AVM, with predominant DAPK/p-DAPK expression in neurons and vasculature. Taken together, these findings suggest DAPK could be a potential molecular target in neuronal death and vascular changes in epilepsy. Increased brain endothelial and astrocytic DAPK in epilepsy, identified for the first time, may have relevance to angiogenesis, hypoxia, and cell survival in pathological conditions.

**Electronic supplementary material:**

The online version of this article (10.1007/s12035-018-1415-z) contains supplementary material, which is available to authorized users.

## Introduction

Neuronal loss after seizure may activate multiple cell death pathways and trigger apoptosis [1]. Scattered evidence in the literature indicates that death-associated protein kinase (DAPK) is correlated with neuronal loss following seizures in rat models [2, 3] and in human epilepsy [4]. However, neurovascular DAPK pathophysiology and its localization in the brains of patients with drug-resistant epilepsy are still unclear. Cortical damage to the drug-resistant epileptic brain due to failed anti-seizure medication and increased seizure frequency could be associated with DAPK cell death.

Recurrent seizures can trigger apoptotic pathways activated primarily during brain development and disease progression [1, 2, 4]. Past evidence already suggests a correlation of DAPK activity with neuronal loss during human brain development, in a process by which more than half the neurons in the brain die before reaching maturation [5, 6]. Furthermore, in developing rats, although DAPK expression was found to be widely distributed throughout the brain, it appeared to be mainly localized in areas exhibiting the greatest changes in neuron density during development, such as the cerebral cortex, hippocampus, and cerebellum [7, 8]. Evidence also indicates DAPK pathway is associated with (1) neuronal stress during oxygen-glucose deprivation condition in ischemia [9] and (2) during angiogenesis affecting vascular endothelial growth factor (VEGF) expression and hypoxia-inducible factor 1α (HIF-1α) activity during tumorigenesis [10].

Since epilepsy and blood-brain barrier (BBB) damage in brain disorders may be life threatening, our aim in the current neurovascular DAPK study was to further elucidate (1) the pattern of localization of DAPK and its phosphorylated form (p-DAPK) in drug-resistant epileptic brain; (2) cellular and subcellular DAPK/p-DAPK levels at the BBB interface in drug-resistant epileptic brain; (3) levels of DAPK, HIF-1α, and VEGF expression at the neurovascular interface in epilepsy compared with other BBB disorders (such as brain tumors [BTs] and arteriovenous malformations [AVMs]); and (4) the effects of DAPK inhibition on p-DAPK, HIF-1α, and VEGF expression and cell viability in human endothelial cells (ECs) and astrocytes obtained from drug-resistant epileptic brains.

## Materials and Methods

### Patient Selection

Brain specimens from patients with temporal lobe epilepsy (TLE), primarily from the cortex region, were obtained for analysis with informed consent in conformity with the principles outlined in the Declaration of Helsinki and institutional guidelines. Additional specimens of brain tumor (BT) and arteriovenous malformation (AVM) were used for comparison. Patient information is summarized in Supplemental Table 1.

### Cell Culture

#### Endothelial Cells

We used primary ECs derived from brain specimens resected from patients with drug-resistant epilepsy (epileptic endothelial cells, EPI-ECs), as described earlier [11, 12]. Briefly, surgical specimens were incubated in collagenase type II (2 mg/ml; Worthington Biochemical Corp., Lakewood, NJ, USA) at 37 °C for 20 min to dissociate the ECs. The collagenase was then washed off with medium (1.5 g/100 ml, MCDB 105 supplemented with EC growth supplement 15 mg/100 ml, heparin 800 U/100 ml, 10% fetal bovine serum, and penicillin/streptomycin 1%). Cells were stained positive for von Willebrand factor and negative for glial fibrillary acidic protein (GFAP). EPI-ECs were initially expanded in 75 cm^2^ flasks pre-coated with fibronectin, 3 μg/cm^2^ [12, 13]. Control human brain microvascular cerebral ECs (HBMECs) were purchased from Cell Systems (Kirkland, WA, USA; catalog number: ACBRI 376). The HBMECs were obtained from different batches to confirm the results obtained from control human brain ECs.

#### Astrocytes

Primary astrocyte cultures (EPI-Astro) were established as previously described [11]. Control human astrocytes (HA) (Cat. 1800) were purchased from ScienCell research laboratories (San Diego, CA, USA) and expanded in poly-d-lysine-coated flask.

We used isolated primary human EPI-ECs (*n* = 5) and EPI-Astro (*n* = 5) from epileptic brain resections and compared them with commercially procured control human brain microvascular endothelial cells (HBMECs, *n* = 5)/astrocytes (HA, *n* = 5) for the following cell culture experiments.

RT-PCR was used to more quantitatively compare the mRNA levels of DAPK in HBMECs, EPI-ECs, HA, and EPI-Astro cells.

### RNA Isolation and Analysis

RNA was isolated via RNeasy Mini Spin Kit (Qiagen); cells (HBMECs, EPI-ECs, HA, and EPI-Astro) were lysed with Qiagen lysis buffer. The cell homogenate was then processed according to an RNeasy Mini Spin Kit protocol (Qiagen); a series of buffers and centrifugations were used to wash the RNA bound to the membrane of the column, with 30 μL RNase free water being used to elute the bound RNA. The RNA extracts were tested via spectrophotometry for purity and concentration, using a cutoff value of 1.8 (Abs 260 nm/Abs 280 nm) to determine acceptable RNA purity.

#### RT-PCR

The Superscript III One-Step RT-PCR with Platinum Taq kit (Invitrogen) was used to prepare all samples. Each sample of RNA was prepared using 1 μg respective RNA isolates, 25 μL 2× Reaction Mix, 0.4 μL each of forward and reverse primers (0.2 μM), magnesium sulfate 10 μL (2 μM), 2 μL Superscript III RT/Platinum Taq Mix, and autoclaved distilled H_2_O to a final volume of 50 μL. Two negative controls were prepared using all reagents except for RNA or enzymes. For DAPK, primer sequences used were forward: TTT TGC TGA AGG CAT CCT CT, reverse GGC AGA GAA ACA GGT CAA GC; for GAPDH forward ATC ACC ATC TTC CAG GAG CG, reverse TTG TCA TAC CAG GAA ATG AG. All samples were placed into an automated thermocycler with an initial cycle of 30 min at 55 °C, followed by 2 min and then 10-s cycles at 95 °C, a 45-s cycle at 58 °C, and then a 90-s cycle at 70 °C. The cycles from 2 min at 95 °C onward were repeated either 35 or 40 times, before a final cycle of 5 min at 70 °C and cooling thereafter. To reduce variation between samples, all samples were reverse transcribed using the same chemicals and enzymes. All PCR products were separated using agarose gel electrophoresis, visualized after staining with ethidium bromide, and the images were captured. Band densities were analyzed with ImageJ software. The band intensity of DAPK for each sample was normalized to the internal standard (glyceraldehyde 3-phosphate dehydrogenase, GAPDH) present in each sample.

### Protein Isolation (Brain Cells and Tissue), Subcellular Fractionation of Brain Tissue, and Western Blotting

Total proteins were extracted from the primary ECs (HBMECs and EPI-ECs) and astrocytic cultures (HA and EPI-Astro) as described previously [12, 14]. To identify the subcellular localization pattern of DAPK and p-DAPK, the subcellular fractions of the resected brain tissue were isolated (cytoplasmic [cyto], mitochondrial [mito], and microsomal [micro]) using a Subcellular Protein Fractionation Kit (Pierce Biotechnology, Inc. [Thermo Scientific], Rockford, IL, USA; cat. 78,833/78835) according to the manufacturer’s guidelines, and the cyto, mito, and microextraction reagents were used to prepare extracts.

#### Western Blot Analysis

Proteins were separated by sodium dodecyl sulfate-polyacrylamide gel electrophoresis and transferred onto polyvinylidene fluoride membranes (EMD Millipore Corp., Billerica, MA, USA). The membranes were probed overnight at 4 °C with the primary antibody (listed in Supplemental Table 2A, which includes the dilution and source) and the appropriate secondary antibody (see Supplemental Table 2B). Polyvinylidene fluoride membranes incubated for 30 min at 50 °C in stripping buffer were later normalized with β-actin used as loading controls. Protein expression was quantified by ImageJ software (National Institutes of Health, Bethesda, MD).

### Histological and Immunohistochemical Staining

We used brain tissues from patients who had undergone surgical resection for intractable epilepsy (*n* = 22), BT (*n* = 4), and AVM (*n* = 3). Control autoptic brain specimens (*n* = 4) obtained from cardiomyopathic subjects (nonneurological case) were used for comparison. Gross anatomical evaluation was made by Cresyl violet staining which was performed on brain slices (*n* = 5 per specimen) for cytoarchitectural analysis to identify dyslamination, ectopic neurons, and vascular malformations [13]. Immunohistochemical staining was performed on successive sections (10 μm; *n* = 5 per specimen, in triplicate) obtained from blocks of resected brain tissues (Supplemental Table 1).

#### Diaminobenzidine and Immunofluorescence Staining

##### DAB Staining

Brain sections were incubated at 4 °C overnight with primary DAPK and p-DAPK antibodies. The detailed method is described previously [12, 13]. After washes, sections were incubated for 1 h at 25 °C with a biotinylated anti-mouse IgG or anti-rabbit IgG based on the species (primary and secondary antibodies used are listed in Supplemental Table 2A and 2B) followed by 1 h with the avidin/biotin complex (Elite Vectastain ABC kit, cat. PK-6102; Vector Labs, Burlingame, CA, USA), visualization with diaminobenzidine (DAB) (peroxidase substrate kit, SK-4100; Vector Labs), dehydration, and mounting.

##### Immunofluorescence

Immunofluorescence staining was performed across brain specimens. These were 10-μm-thick slices, stained/co-stained for DAPK, p-DAPK, HIF-1α, and VEGF. Astrocytic (GFAP) and neuronal (NeuN, neuronal nuclei) markers were also used [13, 14] to confirm the precise cellular localization of the staining. The concentrations and sources of all primary and secondary antibodies used are listed in Supplemental Table 2. In brief, after blocking, sections were incubated in the first primary antibody at 4 °C overnight, followed by 2-h incubation at 25 °C in the corresponding secondary antibody. Sections were then incubated in the second primary antibody at 4 °C overnight, followed by 2-h incubation at 25 °C in the second secondary antibody. Blocking for autofluorescence with Sudan B preceded mounting with VECTASHIELD® Mounting Medium with DAPI (Vector Laboratories, cat. H-1200). Sections were analyzed by fluorescence microscopy and the acquired images were processed using ImageJ software.

### DAPK Inhibition and Immunocytochemical Evaluation

To assess the effects of DAPK inhibition in HBMECs, EPI-ECs, and HA and EPI-astro cells, the cells were cultured in chambered slides and either treated with DAPK inhibitor, (4*Z*)-4-(3-Pyridylmethylene)-2-styryl-oxazol-5-one or not treated; later, DAPK, p-DAPK, HIF-1α, and VEGF expression was evaluated by immunocytochemistry. Cells were plated on fibronectin- or poly-d-lysine-coated chamber slides at a seeding density of 30 × 10^3^ cells/well. Each chamber was separately designated as in the treated or untreated group. A stock of 10 mg DAPK inhibitor (cat. 324788; Calbiochem, now part of Millipore Sigma, Burlington, Massachusetts, USA) was dissolved to make a concentration of 90 mM of stock. In all treated groups, a final concentration of 100 μM of DAPK inhibitor was added for 24 h. The cells incubated with DAPK inhibitors and untreated cells were imaged periodically with an inverted phase microscope (data not shown). The cells (HBMECs, EPI-ECs, HA, and EPI-Astro cells) were subsequently washed, fixed with 2% formalin, washed again with 1× PBS, and then processed by immunofluorescence staining. Primary antibodies (DAPK, p-DAPK, HIF-1α, and VEGF) and respective secondary antibodies used are listed in Supplemental Table 2. The immunocytochemistry procedure described earlier [15] was followed. Cells were visualized using the fluorescence microscope and compared within the DAPK inhibited vs. non-inhibited condition in the ECs and brain astrocytes. Images were subsequently processed using ImageJ (National Institutes of Health, Bethesda, MD, USA) and Q-Capture-Pro™ software (QImaging, Inc., Surrey, BC, Canada). All images were converted to an 8-bit format; brightness/contrast was adjusted using the same parameters for all fluorophores. Threshold signals were set and segmented via the watershed feature of ImageJ. The number of cells per square millimeter intensity was determined in each individual field. Values were collected and expressed as mean ± standard error of the mean.

### Cellular Metabolic Activity Determined by 3-(4,5-Dimethylthiazol-2-Yl)-2,5-Diphenyltetrazolium Bromide (MTT) Analysis

Metabolic activity of endothelial cells (EPI-EC, HBMEC) and astrocytes (Epi-Astro, HA) was determined using MTT assay [16]. Cells were seeded in multiple 12-well plates at a density of 1 × 10^5^ cells per well and left overnight to adhere. DAPK inhibitor (100 μM) was prepared in the respective media for each cell type and incubated for 24 h. Metabolic activity of the cells was assessed by adding 20 μl of MTT (stock 5 mg/ml) for 4 h. Post-incubation, free MTT was removed and 200 μl of DMSO was added to solubilize the purple formazan crystals formed in the wells. Absorbance of the colored complex was assessed using a spectrophotometer (BioTek, Synergy HT, USA) at the dual wavelength 540 and 630 nm using Gen5 software. Metabolic activity was estimated by determining the percentage metabolic or cell viability as described elsewhere [17].

### Adenylate Kinase Measurement

Assessment of cytotoxicity or cell stress was analyzed by estimation of adenylate kinase (AK) [18] release from the endothelial cells (EPI-EC; HBMEC) and astrocytes (EPI-Astro; HA) subsequent to DAPK inhibition. Cells were seeded in multiple 12-well plates at a density of 1 × 10^5^ cells per well and left overnight to adhere. Then DAPK inhibitor (100 μM) was prepared in the respective media and incubated for 24 h. Cell morphology of the brain endothelial and astrocytes were periodically monitored. Media samples were collected before and after the treatment with DAPK inhibitor. The detection was performed with the use of ToxiLight™ Non-destructive Cytotoxicity BioAssay Kit (Lonza, NJ, USA) following manufacturer instructions. The detection reagent was added to the samples, and photon emission indicating the presence of ATP was recorded by spectrophotometer (BioTek, Synergy HT, USA).

### Statistical Analysis

As stated above, all data are expressed as mean ± standard error of the mean. Student’s *t* test was used to compare mean values. Other data were analyzed by one-way analysis of variance (ANOVA) followed by a Bonferroni test for multiple comparisons. A *p* value of < 0.05 was considered statistically significant. Origin 9.0 software (OriginLab Corp., Northampton, MA, USA) was used for statistical analyses.

## Results

### Increased Neurovascular DAPK and p-DAPK Expression and Microsomal Localization Specificity in Epileptic Brain

**Fig. 1:**
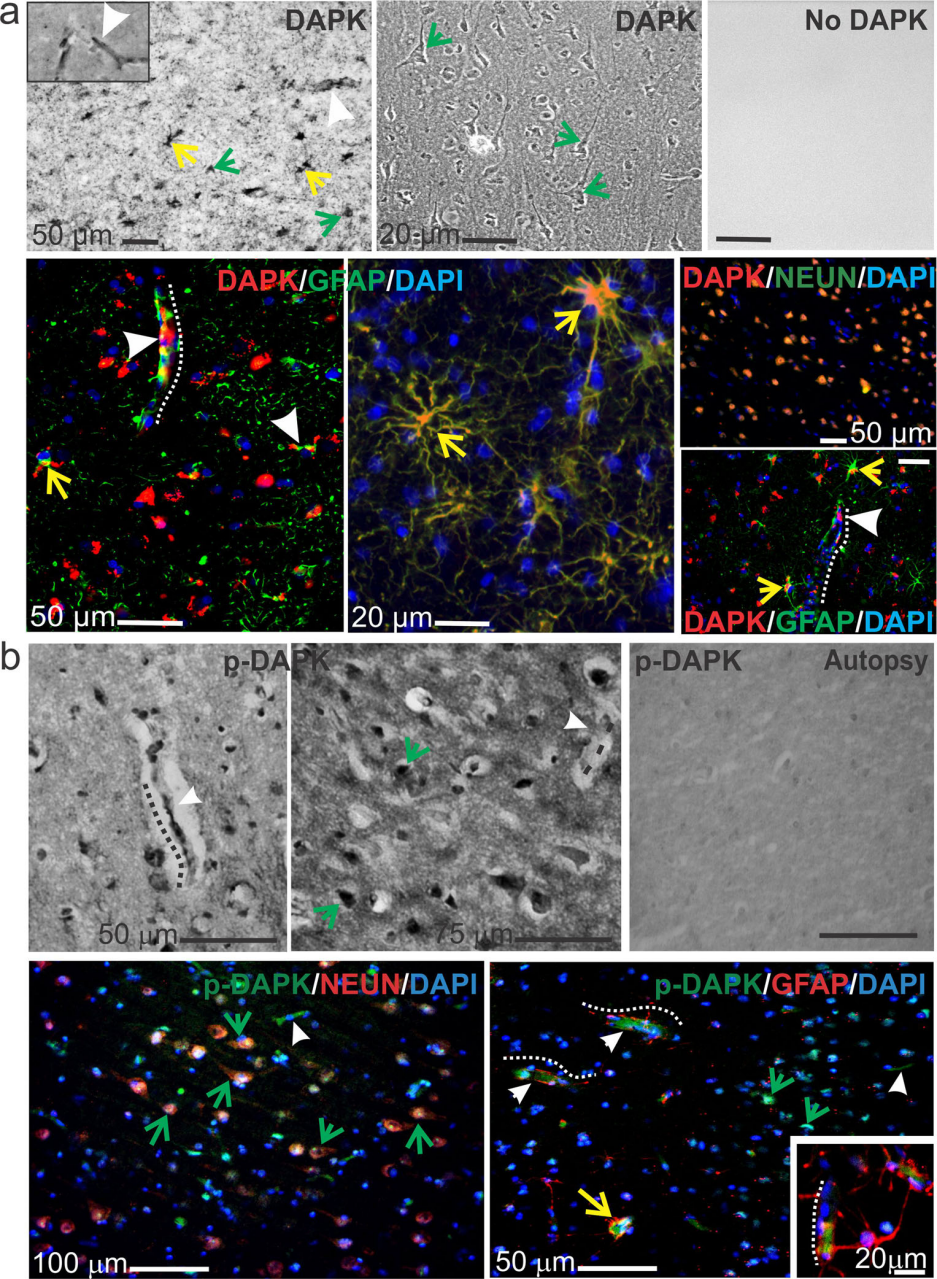
DAPK and p-DAPK expression in the brains of patients with human drug-resistant epilepsy. **a** Temporal lobe epilepsy (TLE) brain resection shows DAPK expressed in ECs, astrocytes, and neurons, highlighted by diaminobenzidine (DAB) staining (top row, with negative control) and immunofluorescence (bottom row). **b** Similarly, increased p-DAPK expression in the ECs and neurons of TLE brain slices was found. *Arrowheads and dotted lines* indicate microcapillaries, *yellow* arrows indicate astrocytes, and *green* arrows indicate neurons. In the autoptic brain tissues (from cardiomyopathy subjects), no specific staining was observed. Note: The standard markers for neuronal nuclei (NeuN) and glial fibrillary actin protein (GFAP) were used

**Fig. 2:**
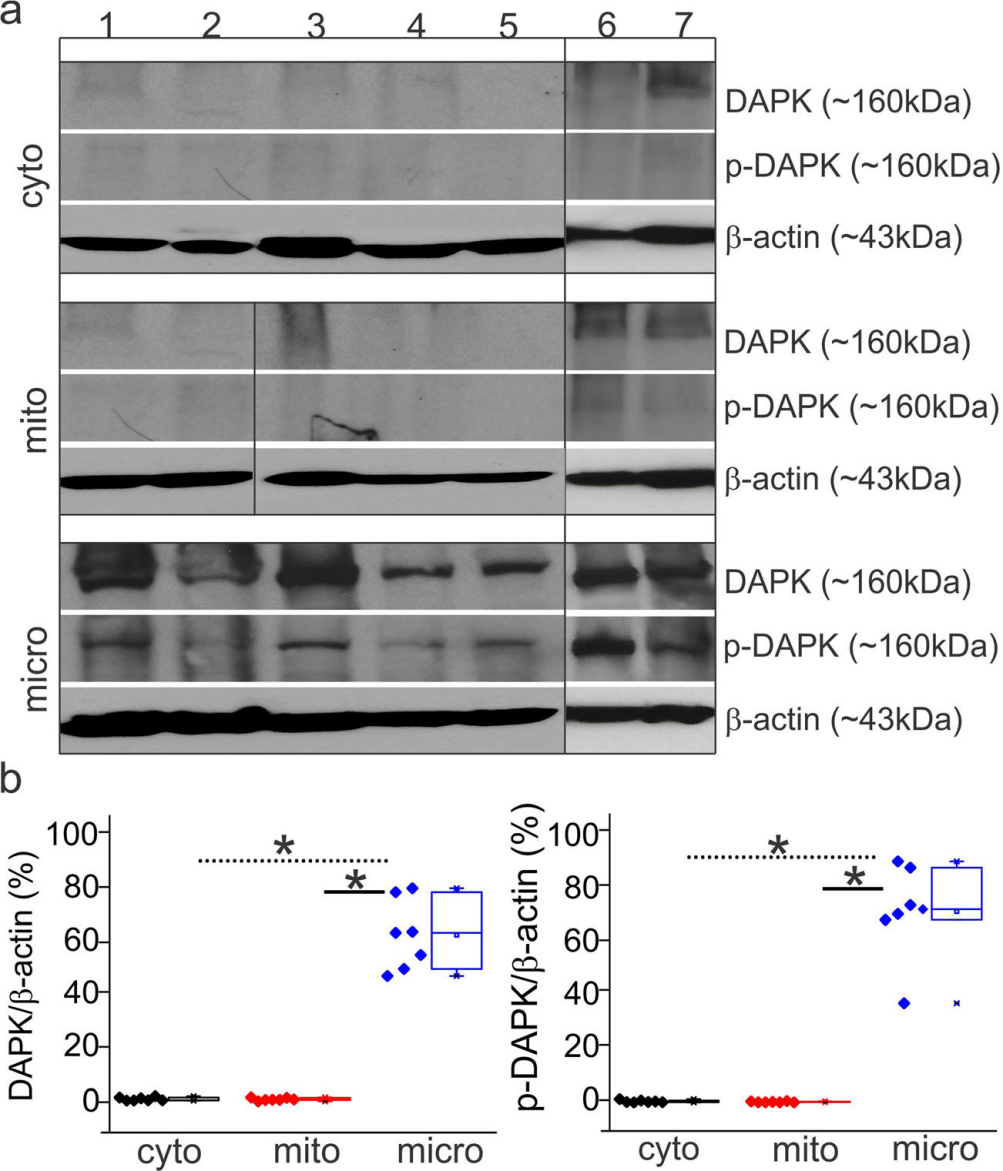
Subcellular localization of DAPK and p-DAPK in the epileptic brain. **a** Western blots of TLE brain specimens (#1–7) show that DAPK and p-DAPK are both localized in the microsomal (micro) fractions rather than to the cytoplasmic (cyto) or mitochondrial (mito) fraction (*n* = 7), indicating that DAPK activity is centered around the endoplasmic reticulum. **b** The relative ratio of DAPK/p-DAPK to β-actin increases only in the microfraction. Data is expressed as mean ± S.E.M. *p < 0.05. ANOVA, analysis of variance

The staining patterns observed across drug-resistant epileptic brain through immunohistochemistry suggest that DAPK expression becomes localized in the microcapillaries and astrocytes and across neurons (Fig. 1). DAPK staining by immunofluorescence indicates co-localization with the astrocytic marker GFAP, neuronal marker NeuN and microcapillaries (indicated by *white arrowheads*) suggesting the specific neurovascular localization of DAPK in the tissue specimens. Increased p-DAPK expression was observed also in the capillaries (*dotted white lines*), astrocytes (*yellow arrows*), and neurons (*green arrows*) predominantly in the epileptic human brain tissues compared with autopsy (control, nonneurological brain specimen). The subcellular fractionation of the epileptic brain tissue by western blotting performed on these drug-resistant epilepsy specimens (7/7 subjects; Fig. 2a) suggests increased expression of DAPK and p-DAPK exclusively in the microsomal vs. cytoplasmic and mitochondrial brain tissue fractions. Microlocalization, specificity, and quantification of DAPK and p-DAPK expression (Fig. 2a, b) after subcellular fractionalization of epileptic brain confirm a significant increase in DAPK and p-DAPK expression in the microfractions. We observed only negligible DAPK/p-DAPK levels in the cytoplasmic and mitochondrial fractions of the same epileptic brain tissue. Furthermore, we found no notable difference in overall DAPK or p-DAPK protein or mRNA expression with variation in age or gender by either immunohistochemistry or western blot.

### DAPK and p-DAPK Expression Pattern in Human Epileptic BBB Endothelial Cells and Astrocytes

**Fig. 3:**
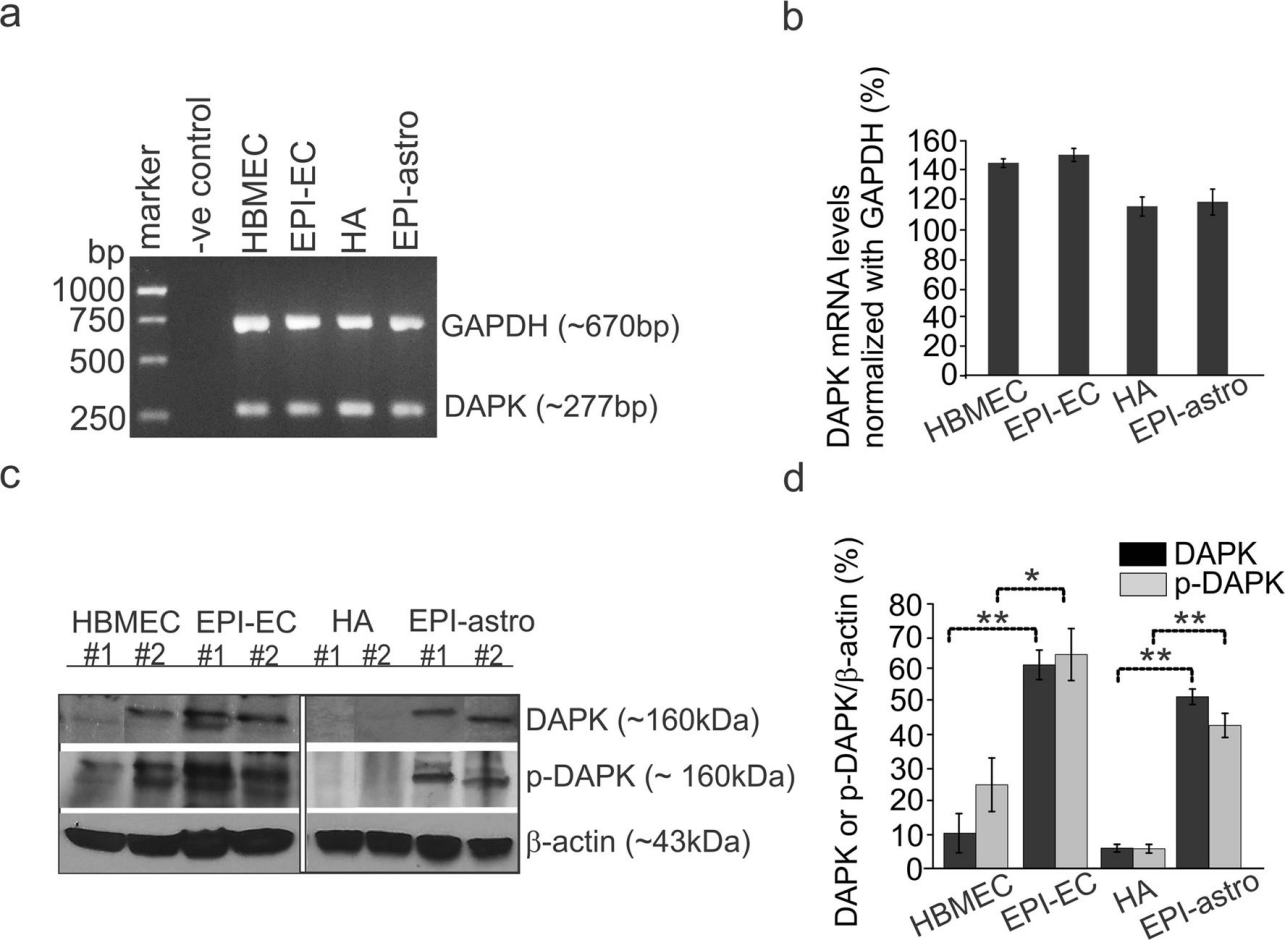
DAPK and p-DAPK expression at human BBB endothelial cells and astrocytes. **a**, **b** DAPK mRNA levels within epilepsy and control, brain endothelial cells, and astrocytes showed no significant difference. However, **c**, **d** DAPK and p-DAPK protein levels were significantly increased in EPI-EC (*p < 0.05, **p < 0.01) compared with those of HBMECs (control, ECs) and EPI-astro cells in comparison to HA (control, astocytes). Data is expressed as mean ± S.E.M. *p < 0.05; **p < 0.01, ANOVA, analysis of variance

DAPK mRNA and protein expression in brain ECs and HA cells (Fig. 3) was determined and compared with cells from normal and epileptic conditions. DAPK mRNA levels showed negligible variability within the normal and epileptic human ECs and astrocytes (Fig. 3a, b); however, the protein expression of DAPK (Fig. 3c, d) was significantly elevated in EPI-ECs compared with normal ones (HBMECs). Similarly, DAPK expression in epileptic astrocytes (EPI-astro) was significantly high compared with normal astrocytes. In addition, levels of p-DAPK were similarly elevated in both EPI-ECs and EPI-Astro compared with normal ECs and normal astrocytes, respectively. Together, these results suggest that human EPI-ECs and EPI-astro cells have increased DAPK and p-DAPK compared with HBMEC or HAs.

### DAPK Inhibition Regulates p-DAPK, HIF-1α, and VEGF Expression in the Endothelial Cells and Astrocytes

**Fig. 4:**
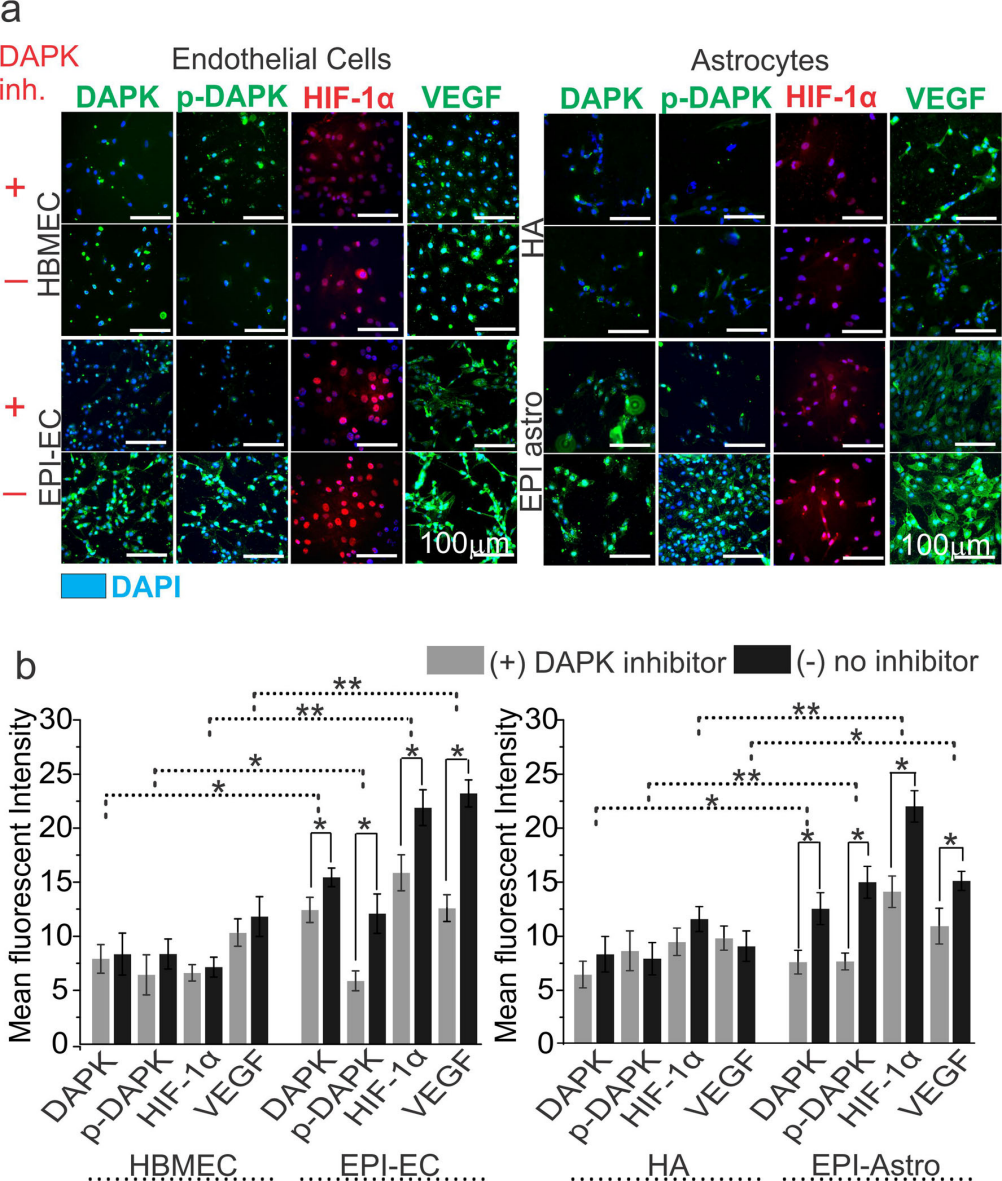
Association of DAPK with p-DAPK, HIF-1α, and VEGF expression in brain endothelial cells (ECs) and astrocytes. **a** Inhibition of DAPK was confirmed by decreased expression of DAPK in the ECs and astrocytes by immunocytochemistry staining. DAPK inhibition caused subsequent inhibition of p-DAPK, HIF-1α, and VEGF expression in the brain ECs and astrocytes. The inhibition was more pronounced in EPI-ECs and EPI-Astro compared to control ECs (HBMECs) and control-Astro cells (HA), respectively. The total number of cells were identified by DAPI-nuclear labelling in blue. **b** Quantification confirms nonsignificant alterations with and without DAPK inhibition on p-DAPK, HIF-1α, and VEGF expression in HBMEC and HA. However, a significant decrease in p-DAPK, HIF-1α, and VEGF levels was observed post DAPK inhibition on EPI-ECs (*p < 0.05). Similarly, EPI-Astro cells showed a significant reduction in p-DAPK and VEGF expression (*p < 0.05) with DAPK inhibition. Consistent elevation in DAPK, p-DAPK, and VEGF expression was found in EPI-ECs and EPI-astro cells compared HBMEC and HAs, respectively without DAPK inhibition. Data is expressed as mean ± S.E.M. *p < 0.05; **p < 0.01, ANOVA, analysis of variance

To further understand the role of DAPK, pharmacological DAPK inhibition [with (4*Z*)-4-(3-pyridylmethylene)-2-styryl-oxazol-5-one] was performed, which successfully decreased DAPK expression in both normal and epileptic human ECs and astrocytes (Fig. 4) in 24 h as confirmed by immunocytochemical staining. Although no significant alteration in the cell morphology was observed, DAPK inhibition resulted in a significant decrease in p-DAPK expression in both EPI-ECs and EPI-astro cells compared with non-DAPK-inhibited conditions in both cell types, suggesting that DAPK regulates p-DAPK expression in both BBB ECs and astrocytes (Fig. 4). Interestingly, DAPK inhibition also resulted in a decrease in VEGF expression in the EPI-ECs and EPI-astro cells compared with HBMEC or HA respectively, thus indicating that the epileptic condition influences DAPK/p-DAPK/ HIF-1α/VEGF expression patterns in BBB ECs and astrocytes. The quantification (Fig. 4b) also confirms an overall increase in DAPK, p-DAPK, HIF-1α, and VEGF expression in EPI-ECs and EPI-astro cells compared with respective controls, i.e., HBMEC and HA, which was significantly decreased (*p < 0.05) post DAPK inhibition.

### DAPK Promotes Cell Survival in Epileptic Brain Endothelial Cells and Astrocytes

**Fig. 5:**
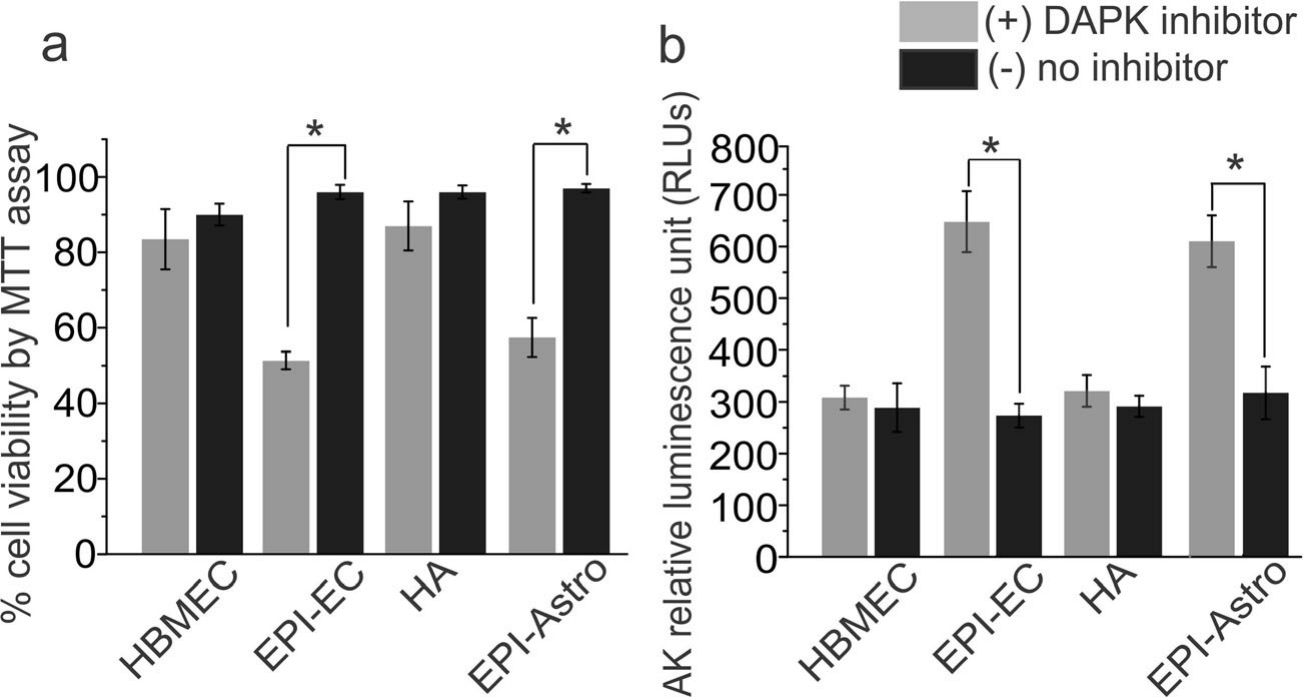
DAPK inhibition regulates cell viability and increases cytotoxicity in human epileptic brain endothelial cells and astrocytes. **a** DAPK inhibition shows significant decrease (*p < 0.05) in cell viability (as percentage) in EPI-ECs and EPI-astro compared to respective untreated cells determined by MTT assay. The control cells (HBMEC and HA) showed negligible alteration. **b** Additional evaluation of cell stress showed cytotoxicity and corresponding increase in AK levels in EPI-ECs (*p < 0.05) and EPI-astro (*p < 0.05) compared to untreated cells subsequent to DAPK inhibition which is indicative of possible involvement of DAPK in cell survival during pathological condition

DAPK inhibition showed cell stress and decreased cell viability significantly in EPI-EC and EPI-Astro (Fig. 5) compared to respective controls (HBMEC and HA). The increased AK levels (*p < 0.05) in EPI-ECs and EPI-Astro was observed post DAPK inhibition indicating cytotoxicity. The levels did not alter remarkably in the HBMEC and HA cells therefore suggesting that DAPK possibly promotes cell survival in the disease state at the brain vasculature.

### Neurovascular DAPK/HIF-1α/VEGF Co-localization Pattern in Epilepsy, Arteriovenous Malformation, and Brain Tumors

**Fig. 6:**
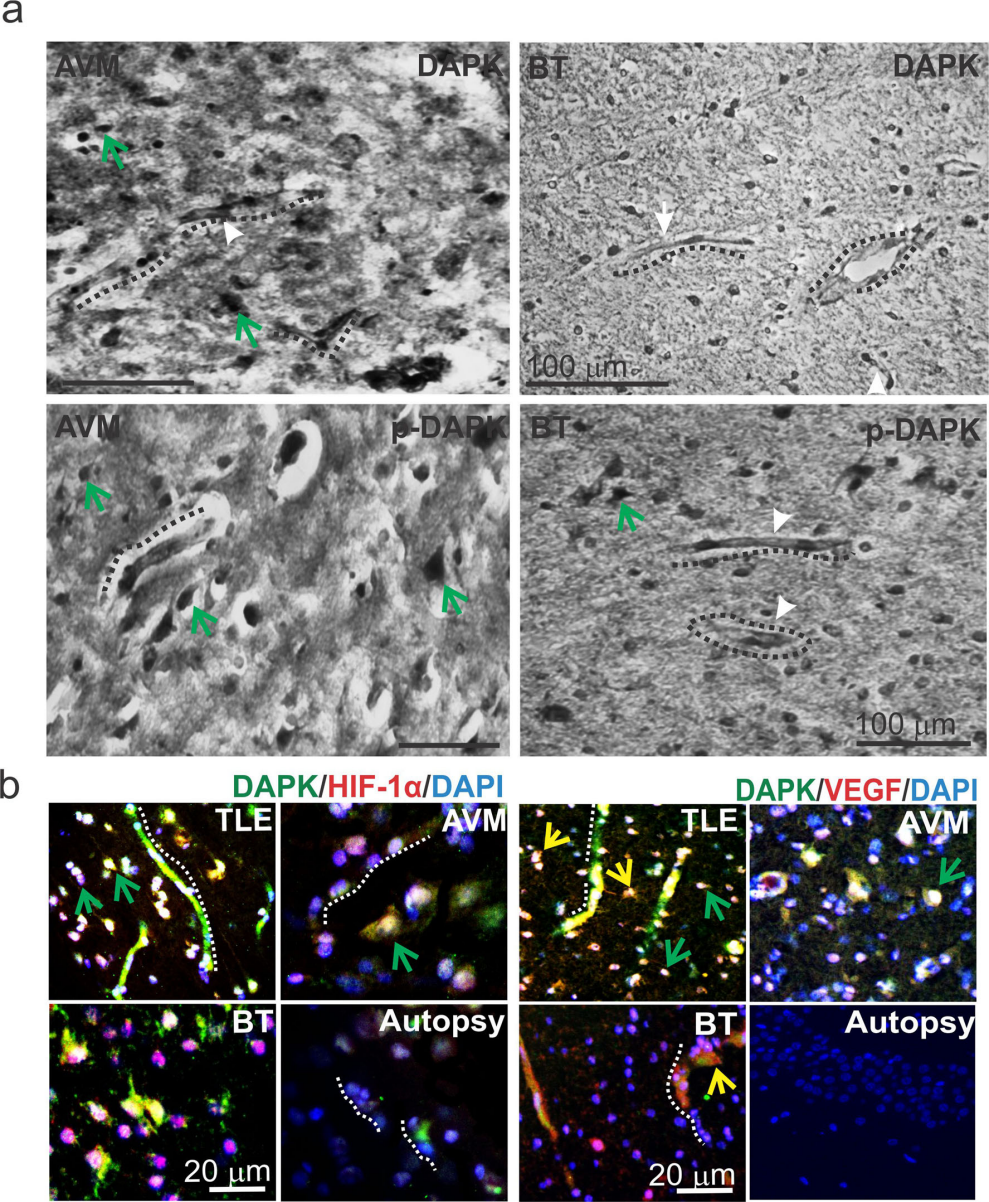
Localization of brain DAPK/p-DAPK expression pattern in AVM and BT and co-localization of DAPK with HIF-1α and VEGF in brain pathologies. **a** DAB staining showed DAPK/p-DAPK localized in the microcapillaries (dotted line) and predominantly across neurons (green arrows) in AVM and brain tumor (BT) specimens. **b** Immunofluorescent staining showed HIF-1α and DAPK increased co-localization predominantly across the neurons in TLE, AVM, and BT compared with autopsy specimens (control); however, TLE showed increased co-staining, which was significantly high in the microcapillaries as well. Elevated p-DAPK and HIF-1α co-staining was found across neurons and capillaries in TLE and AVM brain specimens; however, relatively less staining was evident in BT and autopsy cells. VEGF was co-expressed with DAPK in the TLE brain specimens. Positive staining for VEGF was found mostly in the BBB microvessels, astrocytes (yellow arrow) and limited across the neurons. Increased DAPK and p-DAPK co-expression with VEGF is observed in the brain capillaries and astrocytes in TLE, AVM and BT compared with autopsy samples

Among the brain specimens known to be related with BBB dysfunction such as temporal lobe epilepsy (TLE), AVMs, and BTs, we found increased expression of both DAPK and p-DAPK expression predominantly in the endothelium, astrocytes, and across the neurons (Figs. 1 and 6). Cresyl violet staining (Supplemental Fig. 1) showed neuronal ectopias and dysplastic neurons in TLE, AVM, and BT specimens when compared to samples from autopsy (control brain).

**Fig. 7:**
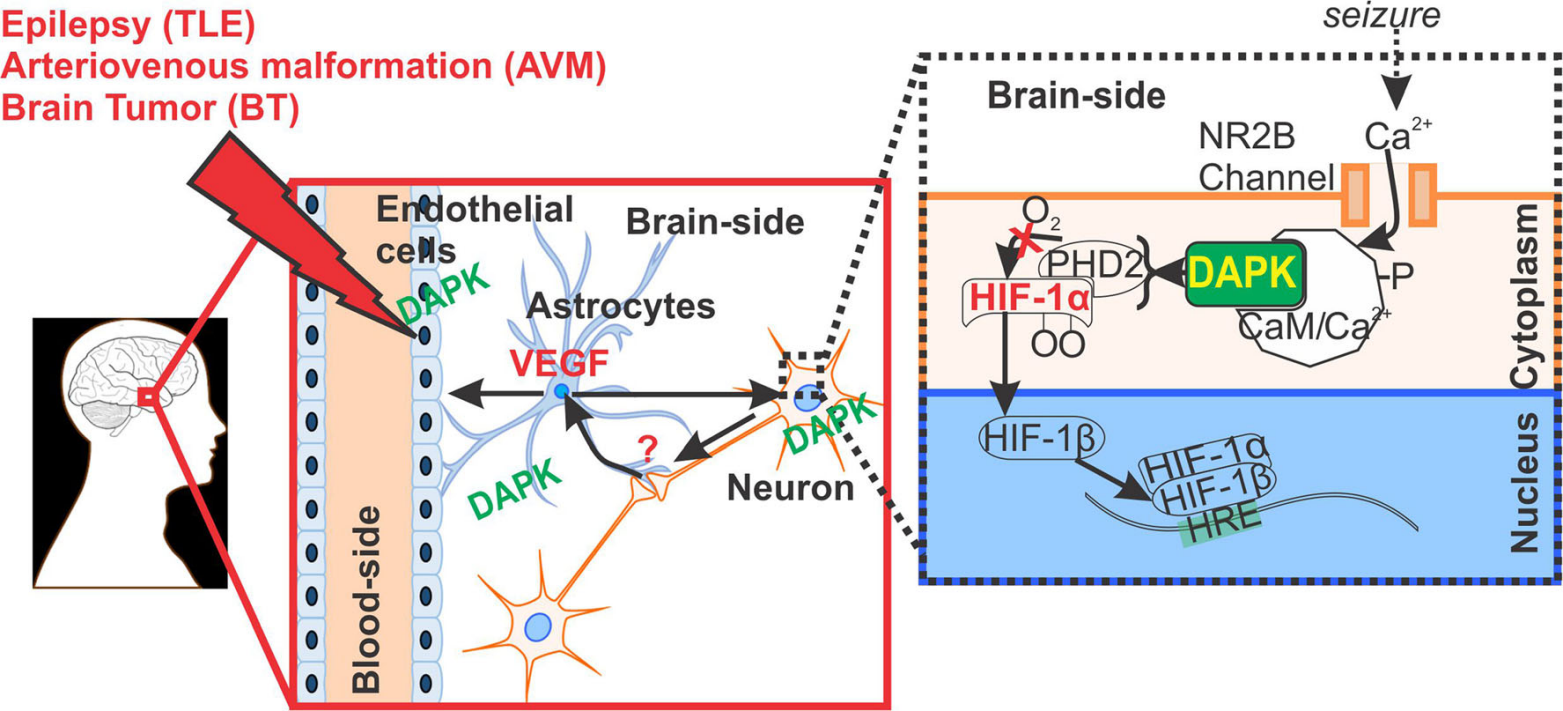
Pathophysiological implication of DAPK regulation at the neurovascular interface. DAPK overexpression identified in the neurons, astrocytes and in brain ECs (EC) microcapillaries may possibly be linked to HIF-1α and VEGF function in brain pathologies triggered by seizures and/or associated with BBB dysfunction. It is known that HIF-1α is activated in ECs and neurons in response to hypoxia [5, 19–21], as shown schematically. Astrocytes receives signals from neurons under hypoxic stress and release VEGF to induce angiogenesis at the neurovascular interface in such brain disorders. DAPK could possibly have a dual role and also indicative of cell survival in disease state (such as epilepsy) cannot be ruled out, which needs further investigation

We also identified (by immunofluorescence) a co-localization of DAPK with HIF-1α extensively across the brain vasculature and neurons in TLE specimens and mostly across the neurons in AVM and BT pathologies compared with autopsy (control). VEGF as a marker of angiogenesis is co-expressed with DAPK more in the microvessels and astrocytes in the TLE and AVM pathologies evaluated. However, limited DAPK was found in the BT pathology when co-labeled with VEGF. Neurovascular DAPK expression and activity could be triggered in brain disorders such as TLE, AVM, and BT by various pathophysiology factors, e.g., seizure, VEGF upregulation, and hypoxic conditions (Fig. 7); these conditions could be regulated by postulating DAPK as a potential therapeutic target for future intervention.

## Discussion

Seizure and progressive brain injury in TLE may trigger several molecular determinants that could identify neuronal and vascular impairment during pharmacoresistance. In the present study, we have established that elevated DAPK and p-DAPK expression in the neurovascular interface could have pathophysiological relevance in drug-resistant epilepsy.

The classical regulatory protein pathway identified earlier is known to be activated during seizure progression. This pathway involves caspase, Bcl-2 proteins, and BAX-1 with pharmacological regulation to prevent seizure and neuronal loss [2, 22–24]. DAPK, a novel calcium-/calmodulin-regulated serine/threonine kinase, is known for its significant features: (1) capability of allowing protein-protein interactions; (2) a death domain, responsible for the assembly of apoptosis-signaling molecules; (3) a kinase domain, which controls catalytic activity; and (4) a cytoskeleton-binding domain, for correct localization of the kinase [5, 25]. Given its ability to interface with proteins related to both apoptosis and cell survival, DAPK may have a significant regulatory role in both processes of the drug-resistant epilepsy brain.

Possible mechanisms of excessive seizures related to neuronal loss may cause cell stress [3]. Excess calcium introduced into the cell can act as a second messenger, binding to calmodulin, which will then allow the phosphorylation of further mediators of apoptosis [5, 26]. In addition to localization of DAPK in the neurons, we found increased expression of both DAPK and p-DAPK immunostaining in the BBB microcapillaries and in astrocytes from the epileptic specimens when compared with control tissue (Fig. 1). However, we identified no notable variation in DAPK or p-DAPK expression between specimens resected from the cortex and hippocampus.

Consistent with earlier reports [4], mitochondria may not be the site of DAPK accumulation in epilepsy, despite the localization of known pro-apoptotic factors to and within the mitochondria/organelle. We found significant elevation of DAPK and p-DAPK exclusively in the microfraction (Fig. 2) compared with the cyto and mito fractions. This explains why the endoplasmic reticulum, a major constituent of the microfraction, is a potent initiator of apoptosis when stressed. Current data and other reports [4, 27, 28] support the significance of DAPK at the endoplasmic reticulum possibly contributing to seizure and drug-resistant epilepsy.

Cellular specificity of DAPK expression in the BBB ECs and astrocytes validates that both brain cells show DAPK mRNA expression, irrespective of control or epileptic cortical origin and a possible posttranscriptional event predominates. Interestingly, overexpression of DAPK protein was more pronounced in the epileptic ECs and astrocytes than HBMEC or HA respectively (Fig. 3). Similarly, p-DAPK expression was elevated in both epileptic tissue and EPI-ECs/EPI-astro cells. Consistent with other reports [4], the increased DAPK phosphorylation in epilepsy specimens that we observed could possibly be related to increased binding with calmodulin and functionally active DAPK.

Besides neuronal injury, hypoxemia is a common occurrence during seizures [29, 30]. Responses to low oxygen concentrations in the brain are coordinated by HIF-1α, a dimeric, oxygen-dependent transcription factor that promotes angiogenesis, vasodilation, and increased ATP production through glycolysis [19, 31]. The angiogenesis regulator VEGF and HIF-1α as a neuronal target in hippocampal sclerosis specimens have been identified [32]. Since BBB damage is associated with HIF-1α and VEGF upregulation [20, 33], we have, for the first time, identified a positive co-localization of HIF-1α and VEGF with DAPK (Fig. 6) in the epilepsy brain vasculature as well as in neurons, which could be due to cerebral apnea and impaired cerebral oxygenation in such brain pathologies. We noticed a close resemblance of the staining pattern of VEGF/HIF-1α/DAPK expression in the epilepsy specimens and other cerebrovascular disorders, such as AVM and BT, suggesting that DAPK expression is correlated to BBB pathophysiology in such brain disorders. Interestingly, the nonneurological specimens (autopsy brain) failed to show this specific staining pattern in the brain. Although the role of VEGF in seizures is unclear [34], it is postulated that VEGF could be responsible for altered BBB permeability, affecting local vascular network, that triggers neuro-inflammatory factors and seizure progression [34–36]. We believe that DAPK plays a major role and acts as a potential therapeutic target. In our current study, further inhibition of DAPK expression (Fig. 4) was found to regulate and decrease the expression of p-DAPK, HIF-1α, and VEGF in both human EPI-ECs/EPI-astro cells obtained from drug-resistant epilepsy, implying a link between DAPK activation, HIF-1α, and VEGF expression. Further, functional assessment by DAPK inhibition may suggest increased cell stress (Fig. 5) with minimal DAPK involvement in human EPI-ECs and EPI-astro cells. Reports also suggest DAPK is a tumor suppressor and mediates degradation of cytoplasmic HIF-1α and a possible treatment strategy for Th17-assocciated inflammatory disease [37, 38]. Therefore, DAPK may be important in promoting cell survival in a pathological state such as epilepsy which needs further investigation.

In contrast to the involvement of neurovascular DAPK with HIF-1α and VEGF in epilepsy according to our current study, the association of DAPK with p53, DANGER, and NMDAR following ischemia and in other neurological disorders has been reported in earlier literature [2, 26, 39]. Evidence also suggests that the conversion of p53 to the functional form *p*S^23^ is mediated by DAPK activity which induces both transcription-mediated apoptosis and mitochondria-associated necrosis in neurons following ischemic insult [2, 39]. Similarly, oxygen-glucose deprivation can increase the association of DAPK with *N*-methyl-d-aspartate receptors, which amplifies their activity and induces toxic calcium influx into neuronal cells [40].

Taken together, the activation and overexpression of neurovascular DAPK identified in human drug-resistant epilepsy (and other neurological conditions) could be a potential molecular target for therapeutic intervention (Fig. 7). Seizure progression and neuronal stress during epilepsy triggers the DAPK pathways, which regulate HIF-1α and angiogenesis genes and proteins (e.g., VEGF) and other factors responsible for BBB function in health and disease.

## Electronic Supplementary Material

Supplemental Fig. 1 Cresyl violet staining of brain pathologies. Representative brain specimens from TLE, AVM and BT sections with Cresyl violet staining are shown. Dysplastic neurons in TLE and AVM pathologies were observed throughout the slices; however, besides the dysmorphic neurons, BT samples also showed shrunken granule cells. A comparatively proper organization of neurons and microvessels is observed in an autoptic brain section (normal brain) (JPG 653 kb)

Supplemental Table 1 Demographic details (DOCX 17 kb)

Supplemental Table 2 List of antibodies used for immunohistochemistry; immunocytochemistry and western blot (DOCX 16 kb)
