# Supplementary figures and images for: Expression and Functional Relevance of Death-Associated Protein Kinase in Human Drug-Resistant Epileptic Brain: Focusing on the Neurovascular Interface
Source: Mol Neurobiol. 2018 Nov 9;56(7):4904–15. doi: 10.1007/s12035-018-1415-z (PMC6509023; doi:10.1007/s12035-018-1415-z)

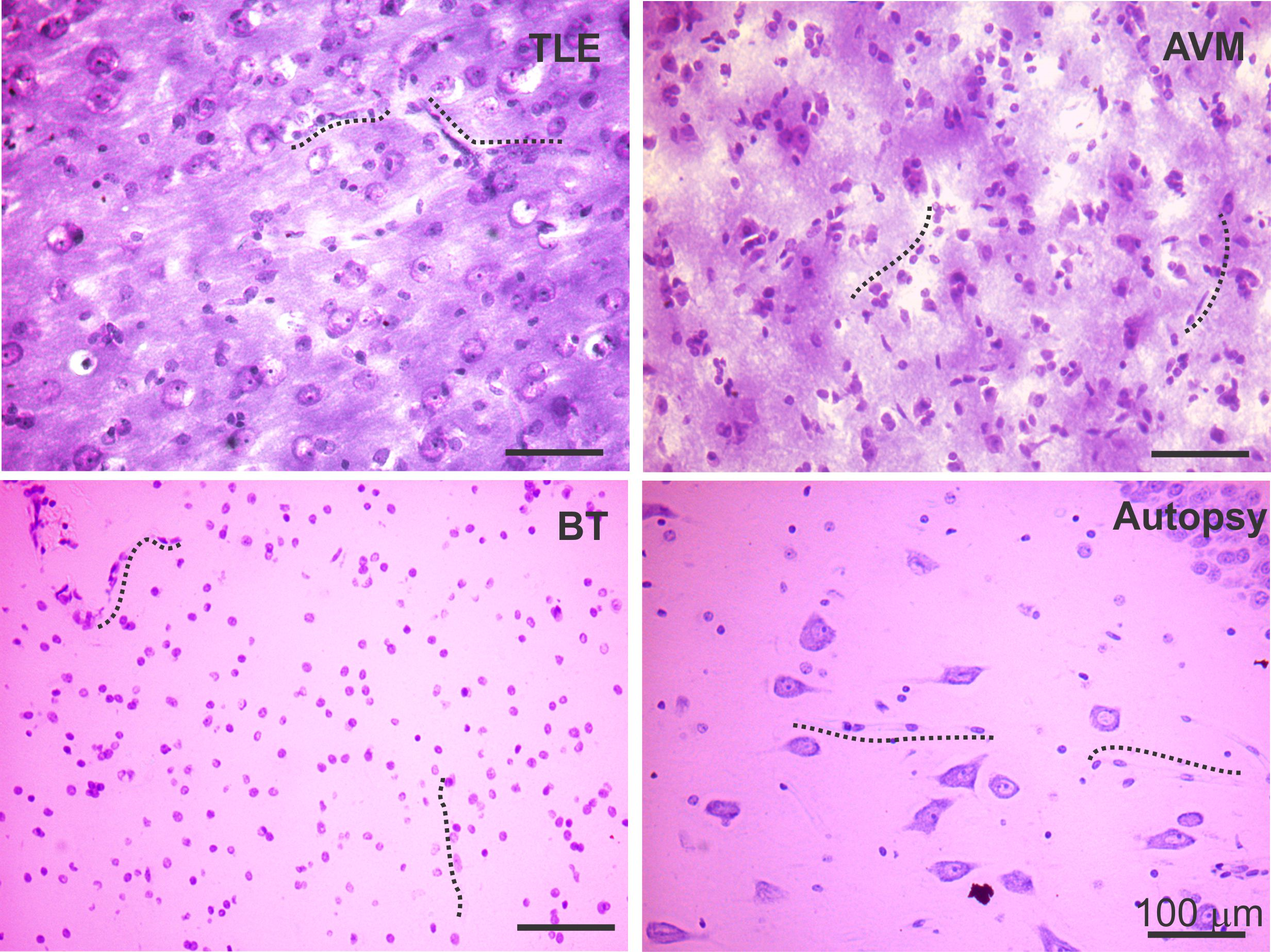

Supplement: Supplementary file 1 — Cresyl violet staining of brain pathologies. Representative brain specimens from TLE, AVM and BT sections with Cresyl violet staining are shown. Dysplastic neurons in TLE and AVM pathologies were observed throughout the slices; however, besides the dysmorphic neurons, BT samples also showed shrunken granule cells. A comparatively proper organization of neurons and microvessels is observed in an autoptic brain section (normal brain) (JPG 653 kb) [file 12035_2018_1415_Fig8_ESM.png]
