# Supplementary material for: Expression and Functional Relevance of Death-Associated Protein Kinase in Human Drug-Resistant Epileptic Brain: Focusing on the Neurovascular Interface
Source: Mol Neurobiol. 2018 Nov 9;56(7):4904–15. doi: 10.1007/s12035-018-1415-z (PMC6509023; doi:10.1007/s12035-018-1415-z)
Supplement: Supplementary file 2 — Demographic details (DOCX 17 kb) [file 12035_2018_1415_MOESM1_ESM.docx]

**Supplemental Table 1. Demographic details**

| **ID** | **Gender** | **Age (yrs/mos)** | **Race** | **Pathology** | **Experimental Use** |
| --- | --- | --- | --- | --- | --- |
| 1 | M | 35 | Caucasian | TLE | WB; CC |
| 2 | F | 10 months | Caucasian | TLE | WB; CC |
| 3 | M | 6 | Caucasian | TLE | WB |
| 4 | F | 3 | Caucasian | Lateral TLE | WB |
| 5 | M | 42 | Caucasian | Left Lateral TLE | WB |
| 6 | F | 49 | NA | TLE | WB |
| 7 | F | 10 | NA | TLE with Hippocampal Sclerosis | WB |
| 8 | F | 60 | Caucasian | TLE | IHC |
| 9 | F | 45 | African-American | TLE | IHC |
| 10 | F | 3 | Caucasian | TLE | IHC; CC |
| 11 | M | 4 | Caucasian | TLE | IHC |
| 12 | M | 7 | Caucasian | TLE | IHC |
| 13 | F | 58 | Caucasian | Lateral TLE/Hippocampal Intractable Seizure | IHC |
| 14 | M | 5 months | Caucasian | Left Frontal Parietal Seizure | IHC |
| 15 | F | 42 | Caucasian | Left TLE | IHC; CC |
| 16 | M | 42 | Caucasian | Occipital Lobe Epilepsy | IHC; CC |
| 17 | M | 45 | African-American | Right TLE | IHC; CC |
| 18 | F | 14 | Caucasian | TLE | IHC |
| 19 | F | 55 | Unknown | TLE | IHC |
| 20 | F | 6 | Caucasian | TLE | IHC; CC |
| 21 | M | 3 months | NA | Temporal Lobe hemimegalencephaly | CC |
| 22 | M | 53 | Caucasian | Temporal Lobe Intractable Seizure | IHC |
| 23 | M | 25 | Caucasian | BT | IHC |
| 24 | F | NA | Caucasian | BT | IHC |
| 25 | F | 29 | Caucasian | Temporal Lobe Tumor | IHC |
| 26 | M | NA | Caucasian | Temporal Lobe Tumor | IHC |
| 27 | M | 48 | Caucasian | AVM | IHC |
| 28 | F | 24 | NA | AVM | IHC |
| 29 | M | 23 | NA | Left Parietal AVM | IHC |
| 30 | M | 25 | Caucasian | BT | IHC |

Abbreviations: AVM, arteriovenous malformation; BT, brain tumor; CC, cell-culture experiments; TLE, Temporal Lobe Epilepsy; IHC, immunohistochemistry; NA, not available; WB, western-blot
