# Supplementary material for: Expression and Functional Relevance of Death-Associated Protein Kinase in Human Drug-Resistant Epileptic Brain: Focusing on the Neurovascular Interface
Source: Mol Neurobiol. 2018 Nov 9;56(7):4904–15. doi: 10.1007/s12035-018-1415-z (PMC6509023; doi:10.1007/s12035-018-1415-z)
Supplement: Supplementary file 3 — List of antibodies used for immunohistochemistry; immunocytochemistry and western blot (DOCX 16 kb) [file 12035_2018_1415_MOESM2_ESM.docx]

**Supplemental Table 2. List of antibodies used for immunohistochemistry/immunocytochemistry and western blot**

| **A. Primary antibodies:** | | | |
| --- | --- | --- | --- |
| **Antibody** | **Species** | **Dilution** | **Source** |
| DAPK | Rabbit | 1:100 (IHC/ICC);  1:500 (WB) | Sigma-Aldrich (SAB4500620), St. Louis, MO |
| DAPK | Mouse | 1:100 (IHC/ICC) | Sigma-Aldrich (D2178), St. Louis, MO |
| p-DAPK | Rabbit | 1:100 (IHC/ICC) | Biorbyt (orb156534), San Francisco, CA |
| p-DAPK | Mouse | 1:100 (IHC/ICC);  1:500 (WB) | Sigma-Aldrich (D4941), St. Louis, MO |
| HIF-1α | Mouse | 1:100 (IHC/ICC) | BD Biosciences (610959), San Jose, CA |
| VEGF | Mouse | 1:100 (IHC/ICC) | Santa Cruz Biotechnology(sc-7269), Dallas, TX |
| NeuN | Mouse | 1:500 (IHC) | Millipore (MAB377), Burlington, MA |
| NeuN | Rabbit | 1:500 (IHC) | Millipore (ABN78), Burlington, MA |
| GFAP | Mouse | 1:100 (IHC/ICC) | Sigma-Aldrich (G3893), St. Louis, MO |
| GFAP | Rabbit | 1:100 (IHC/ICC) | Sigma-Aldrich (SAB4501162), St. Louis, MO |
| β-actin | Mouse | 1:10,000 (WB) | Sigma-Aldrich (A5441), St. Louis, MO |

| **B. Secondary antibodies:** |  |  |
| --- | --- | --- |
| **Antibody** | **Dilution** | **Source** |
| Biotinylated goat anti-rabbit | 1:600 (IHC) | Vector Laboratories (BA-1000), Burlingame, CA |
| Biotinylated goat anti-mouse | 1:600 (IHC) | Vector Laboratories (BA-9200), Burlingame, CA |
| FITC donkey anti-rabbit | 1:600(IHC/ICC) | Jackson Immunoresearch (711-C95-152), West Grove, PA |
| FITC donkey anti-mouse | 1:600(IHC/ICC) | Jackson Immunoresearch (715-095-150), West Grove, PA |
| Alexa 594 donkey α rabbit | 1:600(IHC/ICC) | Jackson Immunoresearch (711-585-152), West Grove, PA |
| Alexa 594 donkey α mouse | 1:600(IHC/ICC) | Jackson Immunoresearch (715-585-150), West Grove, PA |
| Goat anti-rabbit immunoglobulins HRP | 1:2,000 (WB) | Dako, Carpinteria, CA |
| Goat anti-mouse immunoglobulins HRP | 1:2,000 (WB) | Dako, Carpinteria, CA |

Abbreviations: HIF-1α, hypoxia-inducing factor-1α; GFAP, glial fibrillary acidic protein; HRP, horseradish peroxidase; IHC, immunohistochemistry; ICC, immunocytochemistry; NeuN, neuronal nuclei; VEGF, vascular endothelial growth factor; WB, western blot.
